# Characterization of ocular biometrics and aqueous humor dynamics in primary angle closure suspects

**DOI:** 10.1097/MD.0000000000006096

**Published:** 2017-02-17

**Authors:** Li Guo, Yuan Deng, Li Fang, Chaoqi Liu, Tao Guo

**Affiliations:** a Department of Ophthalmology, Shanghai Ninth People's Hospital, Shanghai JiaoTong University School of Medicine; b Department of Ophthalmology, Shanghai Tenth People's Hospital, Tongji University School of Medicine, Shanghai; c Bengbu Medical College, Bengbu, Anhui Province, P.R. China.

**Keywords:** aqueous humor dynamics, intraocular pressure, outflow facility, primary angle closure suspect

## Abstract

Detailed characterizations of ocular biometrics and parameters of aqueous humor dynamics are lacking in primary angle closure suspect (PACS) patients. This study aims to characterize these parameters and compare them with age-matched healthy volunteers.

Elderly healthy volunteers (60.6 ± 7.2 years of age, mean ± SD, n = 28) and PACS patients (64.1 ± 11.6 years, n = 30) completed the study. Parameters investigated were axial length (AXL), anterior chamber depth (ACD), anterior chamber volume (ACV), central cornea thickness (CCT), intraocular pressure (IOP), aqueous flow (Fa), outflow facility (C), episcleral venous pressure (EVP), and uveoscleral outflow (Fu). Comparisons and correlations were made between and within groups.

In healthy volunteers, ocular biometric parameters, IOP, and EVP correlated very well between the 2 eyes of each individual, but Fa, C, and Fu were not significantly correlated. Biometric parameters of the PACS group significantly (*P* < 0.001) differed from those of the healthy controls: AXL (23.31 ± 1.03 mm [PACS] vs 22.39 ± 1.04 mm [Control]; mean ± SD), ACD (2.44 ± 0.33 mm [PACS] vs 1.86 ± 0.25 mm [Control]), ACV (136.0 ± 36.1 μL [PACS] vs 81.4 ± 21.8 μL [Control]), CCT (526.9 ± 37.0 μm [PACS] vs 556.1 ± 28.4 μm [Control]). There was no significant change in IOP, Fa, C, EVP, or Fu between Control and PACS. Furthermore, IOP showed no significant correlations with AXL, ACD, ACV, CCT, or C in both groups.

The PACS eyes had a shorter AXL, a shallower ACD, and a smaller ACV, but a thicker CCT. Despite these morphological changes, the PACS eyes did not have any significant changes in IOP, and aqueous humor dynamics parameters. This is consistent with the findings that IOP did not show significant correlations with biometrics, or C.

## 1 Introduction

Asia accounts for 60% of the world's total glaucoma cases and 76% of the world's primary angle closure glaucoma (PACG) cases.^[1]^ By 2020, Asia will have the largest population affected by PACG.^[1,2]^ The eye of a Chinese adult often has a relatively shallow anterior chamber, small radius of corneal curvature, and narrow anterior chamber angle, all of which could affect intraocular pressure (IOP) and explain the relatively high incidence of angle closure glaucoma in this population.^[3–5]^

The relationship between aqueous humor production and drainage as a function of IOP is described by aqueous humor dynamics (AHD).^[6,7]^ PACG exists in a spectrum of angle closure disorders that includes primary angle closure suspect (PACS), primary angle closure (PAC), and PACG itself.^[8]^ Understanding AHD in PACS sets a foundation for better understanding disease states like glaucoma. Pathological IOP results from a decrease in trabecular outflow facility and in some conditions, a decrease in uveoscleral outflow. IOP also can be raised in disorders that increase downstream resistance in collector channels and episcleral veins.^[9,10]^ Ocular biometric traits like anterior chamber depth, which can influence IOP, are genetically heritable and can vary widely among ethnic groups.^[11]^ It is known that Chinese and certain other Asian people, females, and aged adults have a higher probability of developing angle closure.^[12]^ Eyes with angle closure tend to have a short axial length, a shallow anterior chamber, as well as a thicker and more anteriorly positioned lens.^[13]^ Currently, detailed characterizations of ocular biometric and AHD parameters in the PACG patients are not available. This study assessed and analyzed the differences in aqueous humor dynamics of healthy Chinese elderly adults and PACG patients.

## 2 Methods

### 2.1 Ethical issues

This study adhered to the tenets of the Helsinki Declaration, and was approved by the Research Ethics Committee of the Shanghai Tenth People's Hospital, Tongji University School of Medicine, and all participants have signed an informed consent form.

### 2.2 Inclusion and exclusion criteria

Sixty-two Chinese volunteers were recruited. Voluntary informed consent was procured before the start of any study-related activity. Participants were classified into 2 groups: healthy controls (Control group) and patients with a PACS eye (PACS group). Inclusion and exclusion criteria of the Control group were volunteers with no ocular diseases, best corrected visual acuity better than 20/60, IOP at screening between 12 and 21 mm Hg by a noncontact tonometer (NCT). Exclusion criteria included history of uveitis, ocular trauma, intraocular or refractive surgery, ocular infection within 3 months of enrollment, anterior chamber angles less than Becker–Shaffer grade III,^[14]^ use of systemic medication that affects aqueous humor production such as β-blockers and acetazolamide, history of allergy or hypersensitivity to fluorescein, any abnormalities preventing reliable IOP or fluorophotometric readings, and serious cardiovascular or respiratory diseases. For the PACS group, inclusion criteria were that the patients had been diagnosed with a monocular acute primary angle closure or acute primary angle closure glaucoma, while the contralateral eye had an anatomically narrow angle (less than Becker–Shaffer grade III) but normal IOP (12–21 mm Hg by a NCT), and normal appearance of the optic disc. The eye with these characteristics was defined as PACS eye. Other exclusion criteria were similar to the Control group.

Four volunteers in the Control group chose not to complete all measurements due to discomfort. Two of the PACS patients could not complete all measurements due to equipment failure. The demographic characteristics of the study population who completed the study are summarized in Table 1.

**Table 1:**
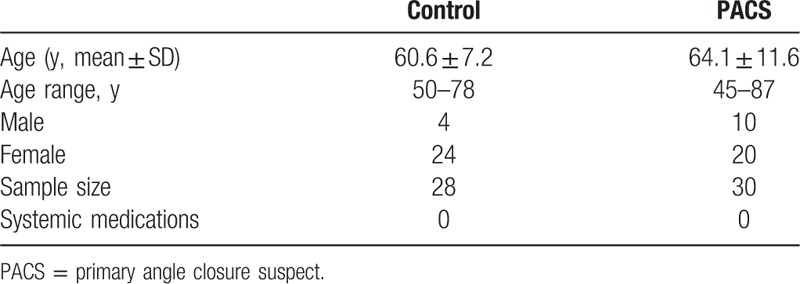
Demographic characteristics of the study participants.

### 2.3 Study design and measurement procedures

**Figure 1:**
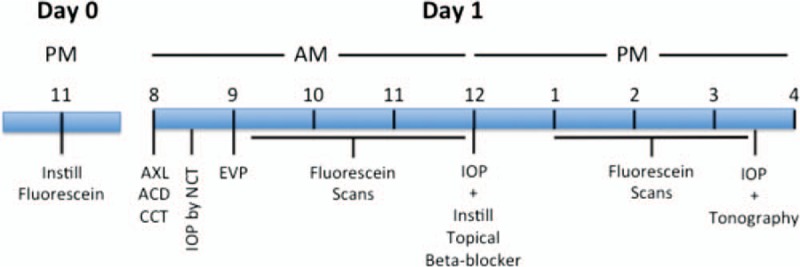
Study procedures and timeline of assessments.

The study procedures and timeline of assessments are shown in Fig. 1. Briefly, volunteers self-administered 8 to 10 drops of 2% fluorescein sodium to each eye starting at 11 pm the night (Day 0) before the study day. Each drop application was separated in time by 5 minutes. All study-related measurements commenced at approximately 8 am the following morning (Day 1) at the eye clinic. Central corneal thickness (CCT) was measured by ultrasound pachymetry (SP-3000, Tomey Corporation Inc, Nagoya, Japan). Anterior chamber depth (ACD) and axial length (AXL) were measured by IOL Master (IOL Master 500; Carl Zeiss Meditec Inc, Dublin, CA). ACD was used in the calculation of anterior chamber volume (ACV).^[15]^ The first IOP was measured at approximately 8:30 am by NCT (CT-1, Topcon, Tokyo, Japan). All subsequent IOP measurements (at 12:30 and 3:30 pm) were made by pneumatonometry (Classic Model 30, Reichert, Depew, NY). Episcleral venous pressure (EVP) was measured with a venomanometer (Eyetech, Morton Grove, IL)^[5]^ after the first IOP measurement. A scanning ocular fluorophotometer (Fluorotron Master; OcuMetrics, Mountain View, CA) was used to measure the intensity of fluorescein in the cornea and anterior chamber. Four sets of fluorescein scans were made in duplicate at intervals of 45 minutes. Aqueous flow (Fa) was determined from the collected scans using the software provided with the Fluorotron. At noon, 1 drop of 0.5% timolol maleate or 0.5% betaxolol was placed in each eye by the investigator. (The first 2 volunteers were given betaxolol and all subsequent volunteers were given timolol.) These drugs lowered IOP by slowing aqueous flow.^[16]^ One hour after dosing with the beta blocker, 3 more sets of fluorescence scans and IOPs were collected. Fluorophotometric outflow facility (Cfl) was calculated for each of the 3 post-drug intervention periods with the formula: Cflx = (aqueous flow − aqueous flowx)/(IOP − IOPx), where aqueous flow and IOP were the baseline values and “x” indicates the corresponding values for the time period (either 1, 2, or 3) after administration of timolol or betaxolol. If aqueous flow or IOP did not decrease during time periods 1, 2, or 3, or were more than 2 standard deviations away from the study mean, Cflx was not calculated for that period. The means of the successfully calculated fluorophotometric outflow facility values at periods 1, 2, and 3 were averaged to yield the reported Cfl. At approximately 3:30 pm, a 2 minute tonography measurement (Cton) was made using the tonography setting on the pneumatonometer. During Day 1, the subjects were permitted to follow their usual dietary habits.

Uveoscleral outflow (Fu) was calculated using Goldmann equation:

Fu = Fa − C (IOP − EVP)

### 2.4 Statistical analyses

The number of subjects enrolled in this study provided a power of 0.95 to detect a 50% difference in outflow facility between groups. Values of Fa, Cfl, and Cton that were above or below 2 standard deviations from the mean were excluded. Data were analyzed using Student 2-tailed *t* test to compare the means of 2 groups using SPSS Statistics 17.0 software. The association between 2 parameters was assessed by linear regression analysis. Data are represented as mean ± SD. Statistical significance was set at *P* < 0.05.

## 3 Results

Fifty-eight volunteers completed the study. Measurements from 28 healthy Control volunteers and 30 patient volunteers were analyzed. The ages of the volunteers ranged from 50 to 78 years (60.6 ± 7.2) and 45 to 87 years (64.1 ± 11.6). The gender distribution was not equal, with females the predominant subset in both groups. None of the Control or PACS volunteers was on systemic medications (Table 1).

**Figure 2:**
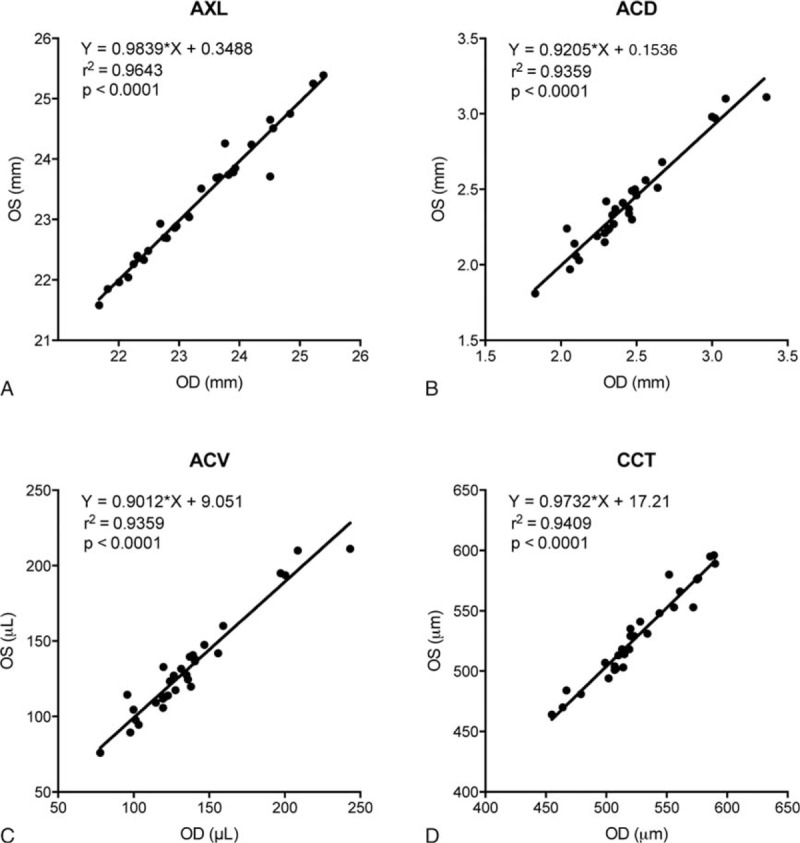
Correlation of biometric parameters between the left (OS) and right (OD) eyes of the Control group. ACD = anterior chamber depth, ACV = anterior chamber volume, AXL = axial length, CCT = central cornea thickness.

**Figure 3:**
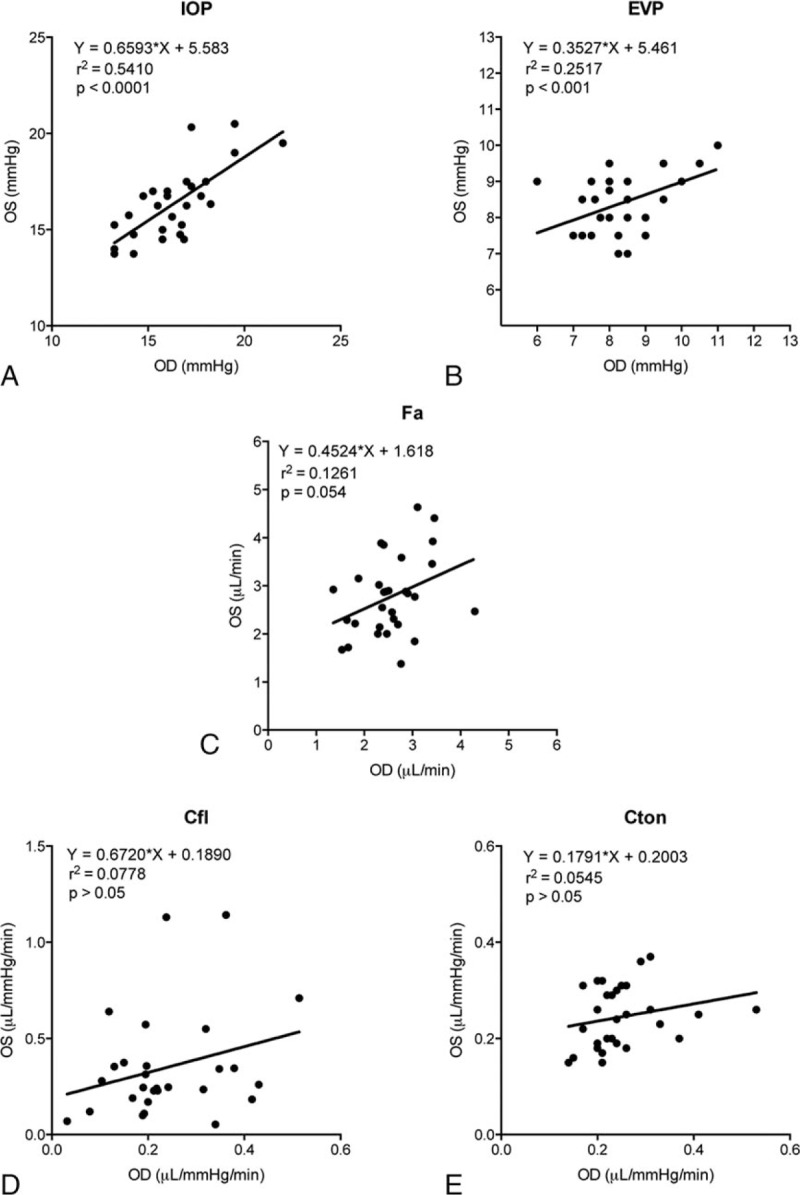
Correlation of aqueous humor dynamics parameters between the left (OS) and right (OD) eyes of the Control group. Cfl = aqueous outflow rate based on fluorescein, Cton = aqueous outflow rate based on tonography, EVP = episcleral venous pressure, Fa = aqueous humor flow, IOP = intraocular pressure.

We compared the biometry of right and left eyes of the Control group. The results indicated that there was excellent (*P* < 0.0001) correlation in biometric parameters: AXL, ACD, ACV, and CCT between the left and right eyes (Fig. 2). Among the AHD parameters, there were significant (*P* < 0.001) correlations between the 2 eyes in IOP and EVP (Fig. 3A and B), but the correlation failed to reach a significant level in Fa, Cfl, or Cton (Fig. 3C–E). The exact reason of this divergence is not clear, but likely partly due to the variances and limitations in precision of the assessment techniques. Since in the majority of cases, the left and right eyes of the same individual correlated well, only the right eyes of members in the Control group were used to compare with the PACS eyes in the following evaluations.

**Figure 4:**
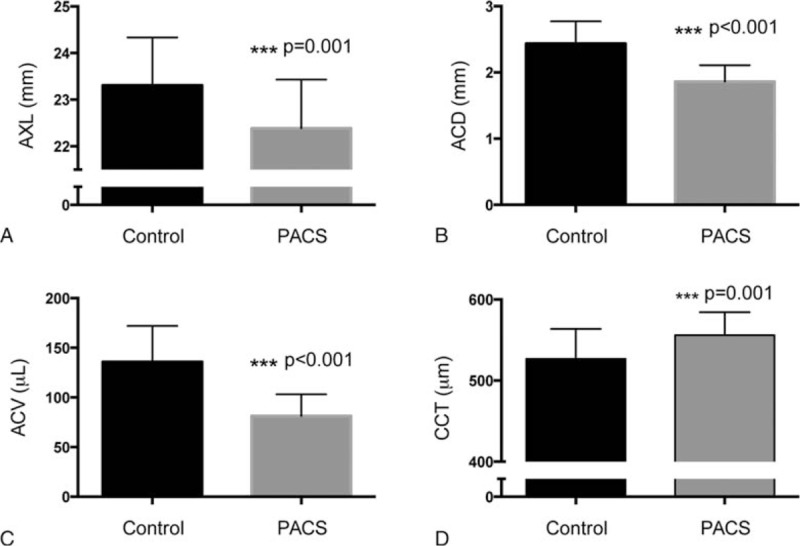
Comparison of biometric parameters between the Control and PACS groups. ACD = anterior chamber depth, ACV = anterior chamber volume, AXL = axial length, CCT = central cornea thickness, PACS = primary angle closure suspect.

Between the Control and PACS groups, there were significant differences in their ocular biometric values. The PACS eyes have a significantly (*P* = 0.001) shorter AXL (22.37 ± 1.06 mm) than the eyes in the Control group (23.34 ± 1.05) (Fig. 4A). Similarly, the ACD was significantly (*P* < 0.001) shallower in the PACS group (1.87 ± 0.24 mm) than the Control group (2.44 ± 0.34 mm) (Fig. 4B). Based on these information, the calculated ACV in the PACS group had a significantly (*P* < 0.001) smaller volume (82.3 ± 21.7 μL) than that of the Control group (136.2 ± 37.5 μL) (Fig. 4C). Interestingly, the PACS group had a significantly (*P* < 0.001) thicker central corneal thickness (555.5 ± 28.6 μm) than the Control eyes (526.2 ± 36.7 μm) (Fig. 4D).

**Figure 5:**
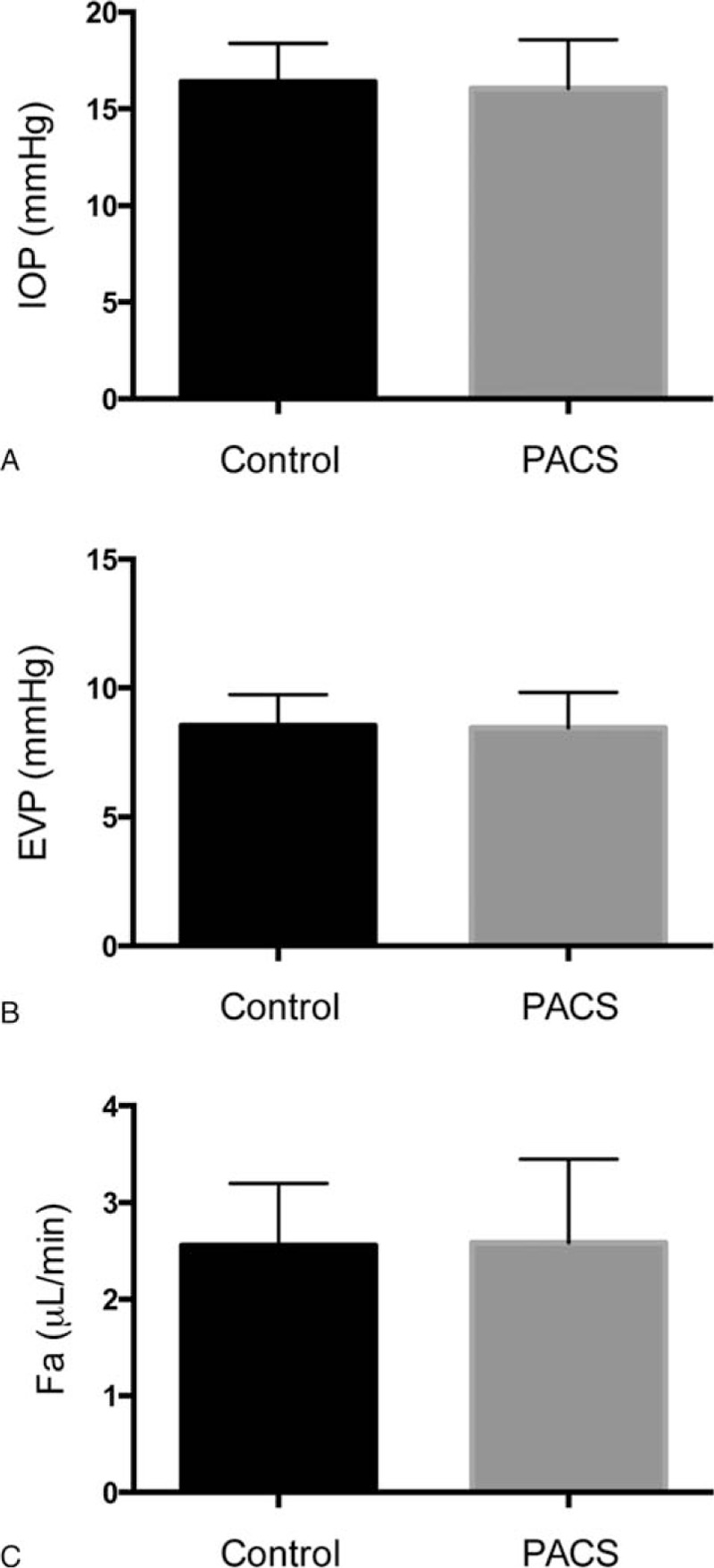
Comparison of IOP, EVP, and Fa between the Control and PACS groups. EVP = episcleral venous pressure, Fa = aqueous humor flow, IOP = intraocular pressure, PACS = primary angle closure suspect.

With the volunteers in the seated position, IOP by NCT (16.4 ± 2.0 mm Hg [Control] vs 16.2 ± 2.5 mm Hg [PACS]) (Fig. 5A), EVP (8.4 ± 1.1 [Control] vs 8.5 ± 1.4 mm Hg [PACS]) (Fig. 5B), and Fa (2.57 ± 0.66 μL/min [Control] vs 2.62 ± 0.86 μL/min [PACS]) (Fig. 5C) were similar between the 2 groups.

**Figure 6:**
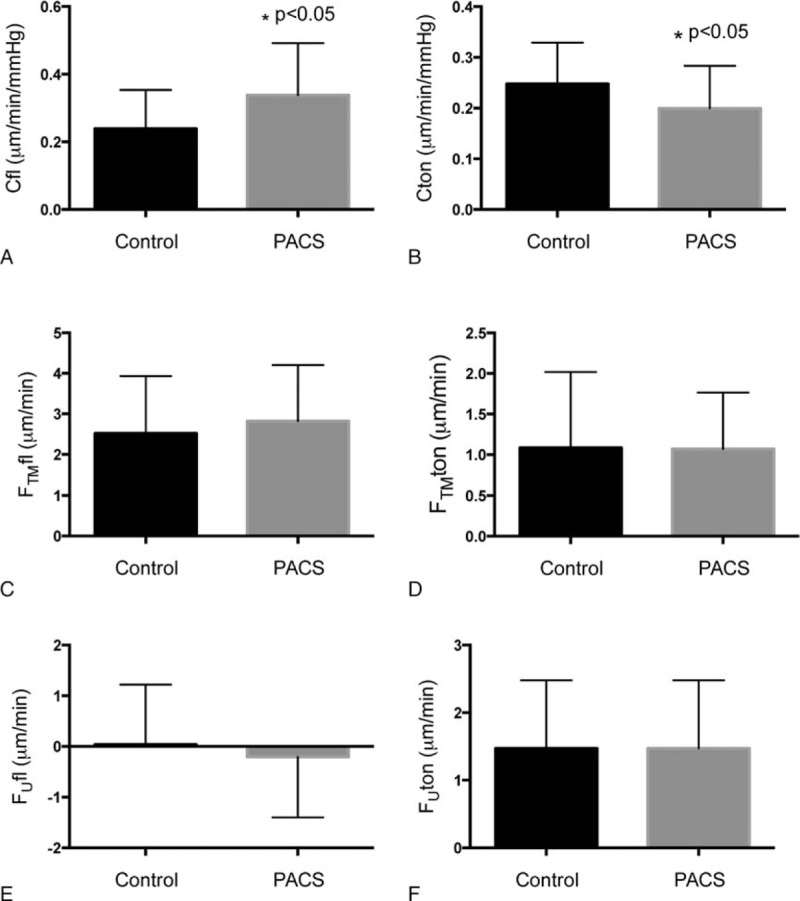
Comparison of outflow parameters between the Control and PACS groups. Cfl = aqueous outflow rate based on fluorescein, Cton = aqueous outflow rate based on tonography, F_TM_fl = conventional outflow rate calculated from Cfl, F_TM_ton = conventional outflow rate calculated from Cton, F_U_fl = uveoscleral outflow rate calculated from Cfl, F_U_ton = uveoscleral outflow rate calculated from Cton, PACS = primary angle closure suspect.

To estimate aqueous outflow facility, we used 2 independent techniques: 1 based on the change in fluorescein-derived flow rates before and after beta-blocker instillation (Cfl), the other based on tonography (Cton). Our results show that Cfl (0.34 ± 0.15 μL/min/mm Hg) in PACS was higher than that in the Control group (0.24 ± 0.11 μL/min/mm Hg) (Fig. 6A), but Cton in PACS (0.20 ± 0.08 μL/min/mm Hg) was lower than that in the Control group (0.25 ± 0.08 μL/min/mm Hg) (Fig. 6B). It has been suggested the shallow anterior chamber in the PACS eyes may interfere with the 2 measurement methods differently, consequently producing different results. Using the C and IOP values, the conventional outflow rates (F_TM_) could be calculated. The flow rate based on Cfl (F_TM_fl) or Cton (F_TM_ton) shows no significant difference between the PACS and Control groups (Fig. 6C and D). Furthermore, the calculated uveoscleral outflow rates, F_U_fl and F_U_ton, were not different between the 2 groups (Fig. 6E and F, *P* > 0.05). These data indicate that the aqueous humor flow rates in PACS patients were similar to those of healthy volunteers.

**Figure 7:**
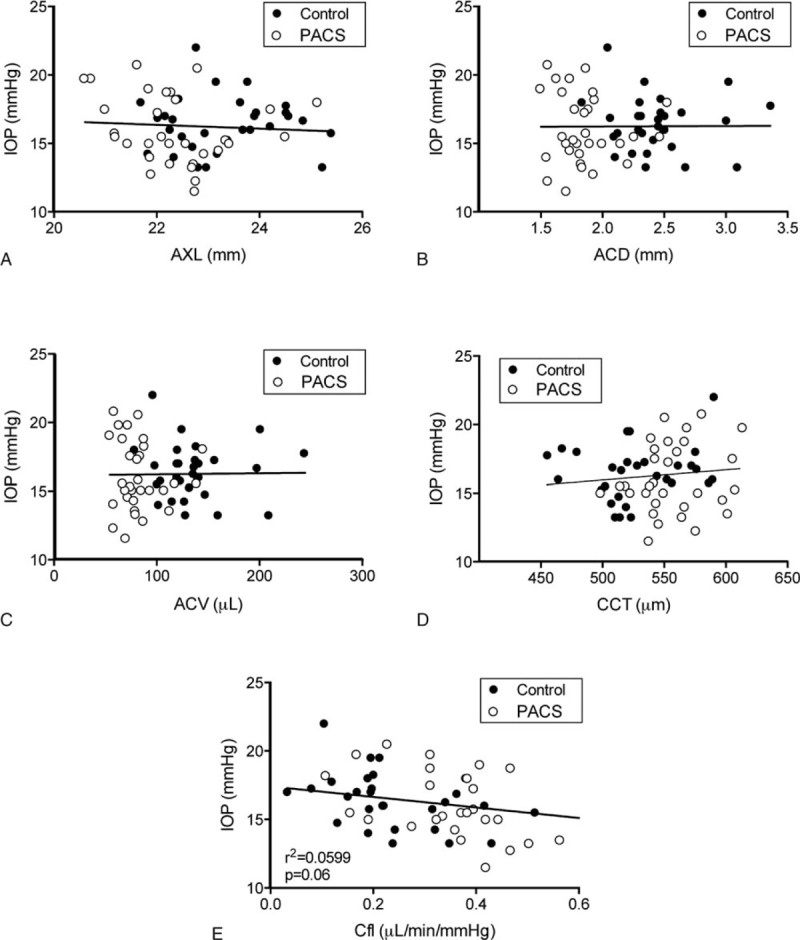
Lack of correlation between IOP and AXL, ACD, ACV, CCT, or Cfl. Data of both study groups were combined. ACD = anterior chamber depth, ACV = anterior chamber volume, AXL = axial length, CCT = central cornea thickness, Cfl = aqueous outflow rate based on fluorescein, IOP = intraocular pressure.

We also evaluated the potential correlation between IOP and the biometrics parameters and Cfl. We did it in separate groups or by combining the 2 study groups, and found that IOP did not significantly (*P* > 0.05) correlate with any of these values. Figure 7 represents analyses by combining both the Control and PACS groups.

## 4 Discussion

The pathophysiology of PACG is not entirely known. However, it is related to abnormalities of the iris, the lens, and structures posterior to the lens. The most common mechanism of angle closure is pupillary block, which creates a significant blockade of aqueous humor flow from the posterior to anterior chambers.^[13]^ The buildup of aqueous humor in the posterior chamber increases the convexity of the iris, effecting angle closure. In Asian patients, other mechanisms unrelated to pupillary block, such as a plateau-like iris configuration, are likely responsible for a significant population of PACG patients.^[17]^

PACG is a family of disorders involving angle closure, such as PACS, PAC, and PACG itself.^[8]^ As observed by gonioscopy, the presence of iridotrabecular contact (ITC) indicates PACS. It is controversial regarding the degree of ITC necessary for a PACS diagnosis, but a majority of ophthalmologists appear to agree that the presence of 180° or more of ITC is sufficient.^[12]^ According to the AAO Preferred Practice Guidelines, 25% patients with PACS eventually develop IOP elevation or peripheral anterior synechiae within 5 years.

In this study, we did not detect differences between healthy adults and PACS in their IOP, EVP, and C, despite the PACS had thicker CCT, shallower ACD, smaller ACV, and shorter AXL. These findings suggest that the morphological changes were not by themselves sufficient to cause changes in AHD or IOP. However, shallow anterior chambers and shorter AXL lead to apposition of the pupil and anterior lens capsule, which can increase the occurrence of pupillary block. In addition, position of the lens plays an important role in the pathogenesis of PACG. Lenses that are more anteriorly positioned cause greater convexity of the iris.^[18]^ In various cases, such as in aged eyes, in phacomorphic glaucoma with advanced cataract, and in instances of choroidal expansion, forward movement of the lens can narrow the anterior chamber and cause contact between iris and trabecular meshwork.^[19]^ Thus, it is very important to continue to monitor these patients.

The principle behind PACG management is to control IOP while monitoring changes to the angle and optic nerve head.^[12]^ Often, this is accomplished by revising the angle configuration through laser/surgical intervention. Aqueous flow was not found to slow significantly with PACS. It is interesting to note that the smaller the anterior chamber depth and AC volume were not consistency with the slower the aqueous flow rate. The uveoscleral outflow shows no significant difference between PACS and healthy group by both fluorophotometry and tonography in our study. Although aqueous suppressants are usually the treatment of choice, prostaglandin analogues increasing in uveoscleral outflow of aqueous humor were recently found effective in lowering IOP in PACG, even in the presence of 360° of peripheral anterior synechiae.^[20]^

## 5 Conclusion

Altogether, the results indicated that there were no significant differences in CCT, ACD, ACV, AXL, IOP, or EVP between right and left eyes in the healthy Control group. ACD, ACV, and AXL decreased, and CCT increased, in the PACS eyes. However, IOP and EVP and other AHD parameters remained unchanged in these patients when compared with the Control group. Also, IOP had no significant correlation with AXL, ACD, ACV, CCT, or Cfl in PACS and healthy groups. This detailed characterization of PACS eyes provides important information about the abnormality.
